# Biometric and ICL-related risk factors associated to sub-optimal vaults in eyes implanted with implantable collamer lenses

**DOI:** 10.1186/s40662-021-00250-6

**Published:** 2021-07-05

**Authors:** Santiago Cerpa Manito, Angel Sánchez Trancón, Oscar Torrado Sierra, António Manuel Baptista, Pedro Miguel Serra

**Affiliations:** 1 Research and Development Department, Ophthalmology Clinic Vista Sánchez Trancón, Building Tecnolaser, Room 14 Calle La Violeta, 06005 Badajoz, Spain; 2 Center of Physics, University of Minho, Braga, Portugal; 3 Optics and Optometry Department, Instituto Superior de Educação e Ciências, Lisbon, Portugal

**Keywords:** Implantable collamer lens, Refractive surgery, Vault, Risk factors

## Abstract

**Background:**

To identify biometric and implantable collamer lens (ICL)-related risk factors associated with sub-optimal postoperative vault in eyes implanted with phakic ICL.

**Methods:**

This study reports a retrospective case series of the first operated eye in 360 patients implanted with myopic spherical or toric ICL. Preoperatively, white-to-white (WTW), central keratometry (Kc) and central corneal thickness (CCT) were measured using the Pentacam. Anterior-segment optical coherence tomography (AS-OCT, Visante) was applied preoperatively for measuring the horizontal anterior-chamber angle-to-angle distance (ATA), internal anterior chamber depth (ACD), crystalline lens rise (CLR), anterior-chamber angle (ACA) and postoperatively the vault. Eyes were divided into three vault groups: low (LVG: ≤ 250 μm), optimal (OVG: > 250 and < 1000 μm) and high (HVG: ≥ 1000 μm). Multinomial logistic regression (MLR) was used to find the sub-optimal vault predictors.

**Results:**

MLR showed that CLR, ICL size minus the ATA (ICL size-ATA), age, ICL spherical equivalent (ICLSE) and ICL size as contributing factors for sub-optimal vaults (pseudo-*R*^*2*^ = 0.40). Increased CLR (OR: 1.01, CI: 1.00–1.01) and less myopic ICLSE (OR: 1.22, CI: 1.07–1.40) were risk factors for low vaults. Larger ICL size-ATA (OR: 41.29, CI: 10.57–161.22) and the 13.7 mm ICL (OR: 7.08, CI: 3.16–15.89) were risk factors for high vaults, whereas less myopic ICLSE (OR: 0.85, CI: 0.76–0.95) and older age (OR: 0.92, CI: 0.88–0.98) were protective factors.

**Conclusion:**

High CLR and low ICLSE were the major risk factors in eyes presenting low vaults. In the opposite direction, ICL size-ATA was the major contributor for high vaults. This relationship was more critical in higher myopic ICLSE, younger eyes and when 13.7 mm ICL were used. The findings show that factors influencing the vault have differentiated weight of influence depending on the type of vault (low, optimal or high).

**Supplementary Information:**

The online version contains supplementary material available at 10.1186/s40662-021-00250-6.

## Background

Implantable collamer lenses (ICL) have been widely used for the correction of high myopia [1], astigmatism [2], and hyperopia [3], with its efficiency and safety demonstrated in various reports (Packer for review) [4]. An implanted ICL is positioned posteriorly to the pupil and rests its haptics on the ciliary sulcus complex. To guarantee a good functional result, the ICL should have minimum interference with the normal physiology of the eye [5, 6]. The vault i.e., the distance between the crystalline lens anterior surface and the ICL, when comprised between 250 to 1000 μm is an indicator of the implant’s safety [7].

Studies reporting on complications associated with earlier ICL versions (e.g., ICL V4b, without a central hole) have found low vaulting as a risk factor for the presence of cataract [8, 9]. The introduction of newer ICL versions with a central hole (e.g., ICL V4c) promotes aqueous humor flow [10] and helps to maintain crystalline lens epithelium cells metabolism [11] which reduces the incidence of cataract [12]. Still, a safety distance between the ICL and the crystalline lens should be maintained since the dynamic behavior of the vault [13] and the age-related thickening of the crystalline lens [14] may lead to the contact of the ICL with the crystalline lens epithelium. The consequences of this contact remain to be studied. On the other hand, high vaults can produce an excessive narrowing of the anterior-chamber angle (ACA) which may hamper the aqueous humor outflow and increase pigment dispersion, and thus place the eye at risk of developing increased intraocular pressure [15]. Furthermore, high vaults may limit pupil dynamics predisposing the patient to the perception of halos [16, 17].

To determine the most appropriate ICL size, various studies have sought to predict the vault of an ICL using preoperative parameters [7, 18–24] showing that the vault is mainly dictated by a relationship between ICL and anatomical factors. Currently, two main principles prevail, one is related to the compression force to which the ICL is exposed due to its larger diameter compared to the transverse size of the eye which produces a forward bulging of the ICL [5, 20]; and the other is associated with the space occupied by the crystalline lens on the sagittal depth of the implanted ICL [7, 21, 25]. The vault predictor models described in the literature [7, 18–24] are based on linear regression analysis assuming a constant relationship between the vault and the independent predictors. However, there is evidence that ICLs implanted with low compression, i.e., small difference between the ICL size and the transverse size of the eye show normal vaults [26]. This leads to the question of whether factors influencing the vault, anatomical and ICL-related, have a differentiated weight of influence depending on the type of vault. Also, the poor performance of some linear regression models points towards the need of better understanding the association between the vault and its predictors [27]. This study aimed to determine the biometric and ICL-related parameters associated with the risk of obtaining a sub-optimal vault i.e., a vault below 250 μm or above 1000 μm. Our findings provide selective information about the anatomical and ICL features regulating low and high vaults which will assist the surgeon in the analysis of the preoperative parameters.

## Methods

### Study design

This study is a retrospective case series of patients implanted with an ICL for the correction of myopia and astigmatism (EVO-V4c, STAAR Surgical Co. Nidau, Switzerland). Patients were operated in the Ophthalmology Clinic Vista Sánchez Trancón (Badajoz, Spain) between 2012 and 2017. The inclusion criteria for the analysis were, spectacle refraction between − 4.00 and − 20.00 diopter sphere (DS), refractive astigmatism lower than − 5.00 diopter cylinder (DC), internal anterior chamber depth (ACD: distance from corneal endothelium to crystalline lens anterior surface) > 2.8 mm and endothelial cell count > 2000 cells/mm^2^. The exclusion criteria were the presence of corneal abnormality such as ectasia, dystrophy or trauma, previous corneal refractive surgery, ICL haptics oriented vertically and eyes with missing or non-suitable pre or postoperative exams. The analyzed sample comprised the first operated eye of 360 patients. The study followed the tenets of the Declaration of Helsinki and gained ethical approval by the local ethics committee (Comité Ético de Investigación Clínica de Badajoz).

### Preoperative and postoperative protocol

The preoperative and postoperative protocols have been described by our group elsewhere [24]. Anterior segment optical coherence tomography (AS-OCT; Visante, Zeiss Meditec AG, Jena, Germany) was used for measuring the horizontal anterior-chamber angle-to-angle distance (ATA), crystalline lens rise (CLR), ACD and the nasal and temporal ACA. The AS-OCT scans were performed along the horizontal meridian using a single-scan centered on the pupil and all parameters were measured using the instrument’s in-built calipers. Scheimpflug photography (Pentacam HR, OCULUS Optikgeräte GmbH**,** Wetzlar, Germany) was used for quantifying the horizontal visible iris diameter (WTW), central keratometry (Kc) and the central corneal thickness (CCT). Endothelial cell count was performed with a noncontact specular microscopy (Topcon SP-2000P, Topcon Corporation, Tokyo, Japan) using a 12-point sampling. All measurements were performed in a room with dim light and the patients were instructed to look at the fixation systems of each instrument. The ICL size and dioptric power were determined using the manufacturer’s calculator.

Postoperative assessment took place on average 15 weeks post-surgery. The central vault was measured using the AS-OCT Visante and defined as the distance between the crystalline lens anterior apex and the most anterior point of the ICL posterior surface.

### Surgical protocol

The surgery was performed under local anesthesia using 2% intracameral Lidocaine (B.Braun® 20 mg/ml). The anterior chamber was filled with viscoelastic (2% methycellulose, Medicontur, Zsámbék, Hungary) and the ICL introduced through a 3.2 mm temporal clear corneal incision using the manufacturer’s injector cartridge (STAAR Surgical Co. Monrovia, CA, USA). Upon positioning the ICL, the viscoelastic was removed through aspiration from the anterior chamber. At the end, a diluted antibiotic solution (Ceftazidime and Vancomicine) was injected. After surgery, antibiotic (Exocin® Ofloxacin 3 mg/ml), corticoid (Predforte® Prednisolone acetate 10 mg/ml) and anti-inflammatory (Voltaren® Diclofenac sodium 1 mg/ml) drugs were prescribed four times a day for 3 weeks.

### Statistical analysis

Preoperative anatomic parameters (ATA, WTW, CCT, ACD, CLR, ACA (average of the nasal and temporal ACA), Kc); demographic (age); ICL properties (ICL size and ICL spherical equivalent (ICLSE)); the difference between the ICL size and the ATA (ICL size-ATA) and the difference between the ICL size and the WTW (ICL size-WTW) were used as predictors of a vault outside an optimal vault range, defined between 250 and 1000 μm. The ICL size-ATA, was also defined as ICL compression [20]. First, the variables in the spherical and toric ICL group were compared using multivariate analysis of variance (MANOVA) to test the hypothesis that the parameters in both ICL groups differed. Three eyes implanted with a 12.1 mm ICL were removed from analysis due to underrepresentation.

Secondly, the sample was divided in three groups according to the vault size; low vault group (LVG: ≤ 250 μm), optimal vault group (OVG: 250 to 1000 μm) and high vault group (HVG: ≥ 1000 μm). Differences among groups were investigated using MANOVA and pairwise comparisons were accounted for by applying the Bonferroni correction. Analysis of covariance (ANCOVA) was used to investigate the influence of ICL size on the type of vault while controlling the ICL size-ATA parameter. A stepwise multinomial logistic regression analysis was used to identify the variables relevant for characterizing the type of vault. The OVG was chosen as the reference group and the 13.2 mm ICL as the reference ICL for ICL size comparison. The statistical analysis was performed using SPSS (IBM®, SPSS® Statistics, v.23).

## Results

Comparison between the spherical and toric ICL groups showed no statistical difference (Pillai’s Trace, F = 1.6, *P* = 0.110), and thus the groups were merged resulting in a total of 360 eyes that were analyzed. Table 1 summarizes the sample details for the two ICL groups.

**Table 1:** Sample demographics summary

Parameter	Spherical		Toric	
N	220		140	
Gender (M/F)	77/143		59/81	
	**Mean**	**95th Percentile**	**Mean**	**95th Percentile**
Age (years)	32.7 ± 7.7	[21.0, 48.0]	31.6 ± 7.3	[21.0, 48.0]
Sphere (DS)	−9.88 ± 3.88	[−20.00, −4.00]	−8.20 ± 3.33	[−15.94, −3.00]
Cylinder (DC)	−0.84 ± 0.70	[−2.00, ±0.00]	−2.71 ± 0.99	[−5.00, −1.27]
CDVA (decimal VA)	0.78 ± 0.39	[0.3, 1.0]	0.76 ± 0.40	[0.3, 1.0]
Endothelial cell count (cells/mm^2^)	2792 ± 382	[2041, 3542]	2879 ± 468	[1757, 3593]
Postoperative follow-up (weeks)	14.0 ± 3.4	[8, 25]	16.0 ± 3.5	[9.0, 27.0]

*CDVA* corrected distance visual acuity; *DC* diopter cylinder; *DS* diopter sphere; *VA* visual acuity

The mean preoperative parameters and postoperative follow-up shown are represented together with the 95th percentile

### Comparison between pre-operative parameters by vault type

Table 2 shows the descriptive statistics for the entire sample and for the three vault groups. Group comparison showed statistically significant differences between the three vault groups (Pillai’s Trace: F = 19.5, *P* < 0.0001). The ICL size, age, ACD, CLR, ICLSE, ICL size-ATA and vault differed among the three vault groups. The remaining parameters were similar among the groups.

**Table 2:** Descriptive statistics for the three vault groups, LVG: ≤ 250 μm, OVG: > 250 and < 1000 μm and HVG: ≥1000 μm

Parameter	All		LVG (≤ 250 μm)		OVG (250 to 1000 μm)		HVG (≥ 1000 μm)		Statistical significance
Gender (M/F)	136/224		14/20		99/173		23/31		*P* > 0.050
ICL size (mm) & number of eyes	12.6	70 (19.4%)	12.6	7 (10.0%)	12.6	58 (82.9%)	12.6	5 (7.1%)	*P* < 0.0001
	13.2	218 (60.5%)	13.2	25 (11.5%)	13.2	169 (77.5%)	13.2	24 (11.0%)	
	13.7	72 (20%)	13.7	2 (2.8%)	13.7	45 (62.5%)	13.7	25 (34.7%)	
	**Mean ± SD**	**95%** **Percentile**	**Mean ± SD**	**95%** **Percentile**	**Mean ± SD**	**95%** **Percentile**	**Mean ± SD**	**95%** **Percentile**	
Age (years)	32.3 ± 7.6	[22.0, 46.0]	35.8 ± 7.4	[25.6, 48.0]	32.5 ± 7.6	[22.0, 46.0]	29.1 ± 6.9	[20.0, 45.25]	*P* < 0.0001
Vault (μm)	660.9 ± 314.6	[170.5, 1230.0]	162.2 ± 65.2	[0.0, 234.0]	619.3 ± 191.9	[310.0, 928.0]	1200.0 ± 161.3	[1000.0, 1580.0]	*P* < 0.0001
WTW (mm)	11.90 ± 0.45	[11.20, 12.60]	11.89 ± 0.41	[11.26, 12.60]	11.84 ± 0.45	[11.10,12.60]	12.02 ± 0.42	[11.30, 12.73]	*P* > 0.050
ATA (mm)	12.20 ± 0.46	[11.46, 12.96]	12.30 ± 0.46	[11.37, 12.96]	12.21 ± 0.46	[11.47, 12.97]	12.17 ± 0.46	[11.29, 12.89]	*P* > 0.050
CCT (μm)	535.2 ± 39.8	[470.0, 610.0]	538.9 ± 39.9	[470.0, 614.0]	534.3 ± 41.0	[470.0, 610.0]	44.17 ± 33.50	[477.5, 595.0]	*P* > 0.050
ACD (mm)	3.29 ± 0.26	[2.88, 3.82]	3.13 ± 0.24	[2.80, 3.55]	3.29 ± 0.25	[2.89, 3.78]	3.42 ± 0.29	[2.90, 3.96]	*P* < 0.0001
CLR (μm)	106.6 ± 209.9	[−240.0, +449.0]	299.7 ± 223.5	[−112.0, +840.0]	95.6 ± 198.8	[−258.0, +418.0]	30.4 ± 178.6	[−225.0, +360.0]	*P* < 0.0001
Kc (D)	43.9 ± 1.7	[40.22, 46.87]	43.9 ± 2.3	[40.2, 46.9]	44.2 ± 1.6	[41.3, 46.4]	44.2 ± 1.9	[40.9, 47.6]	*P* > 0.050
ACA (deg)	41.7 ± 8.1	[29.37, 56.23]	41.0 ± 7.2	[28.4, 56.0]	42.2 ± 8.3	[29.2, 56.5]	42.2 ± 7.8	[29.4, 55.9]	*P* > 0.050
ICLSE (DS)	−10.9 ± 3.3	[−17.97, −16.23]	−9.45 ± 1.87	[−13.60, −6.30]	−11.4 ± 3.36	[−17.50, −7.90]	−11.45 ± 3.64	[−18.00, −6.06]	*P* = 0.015
ICL size-WTW (mm)	1.30 ± 0.34	[0.80, 1.80]	1.21 ± 0.30	[0.76, 1.74]	1.30 ± 0.35	[0.80, 1.80]	1.37 ± 0.30	[0.85, 1.80]	*P* > 0.050
ICL size-ATA (mm)	0.96 ± 0.34	[0.39, 1.49]	0.79 ± 0.37	[0.24, 1.51]	0.93 ± 0.33	[0.39, 1.45]	1.22 ± 0.27	[0.66, 1.66]	*P* < 0.0001

*LVG* low vault group; *OVG* optimal vault group; *HVG* high vault group; *ICL* implantable collamer lens; *WTW* white-to-white (horizontal visible iris diameter); *ATA* horizontal anterior-chamber angle-to-angle distance; *CCT* central corneal thickness; *ACD* internal anterior chamber depth; *CLR* crystalline lens rise; *Kc* central keratometry; *ACA* anterior-chamber angle; *ICLSE* implantable collamer lens spherical equivalent; *DS* diopter sphere. The mean and standard deviation (SD) are represented together with the 95th percentile

**Fig. 1:**
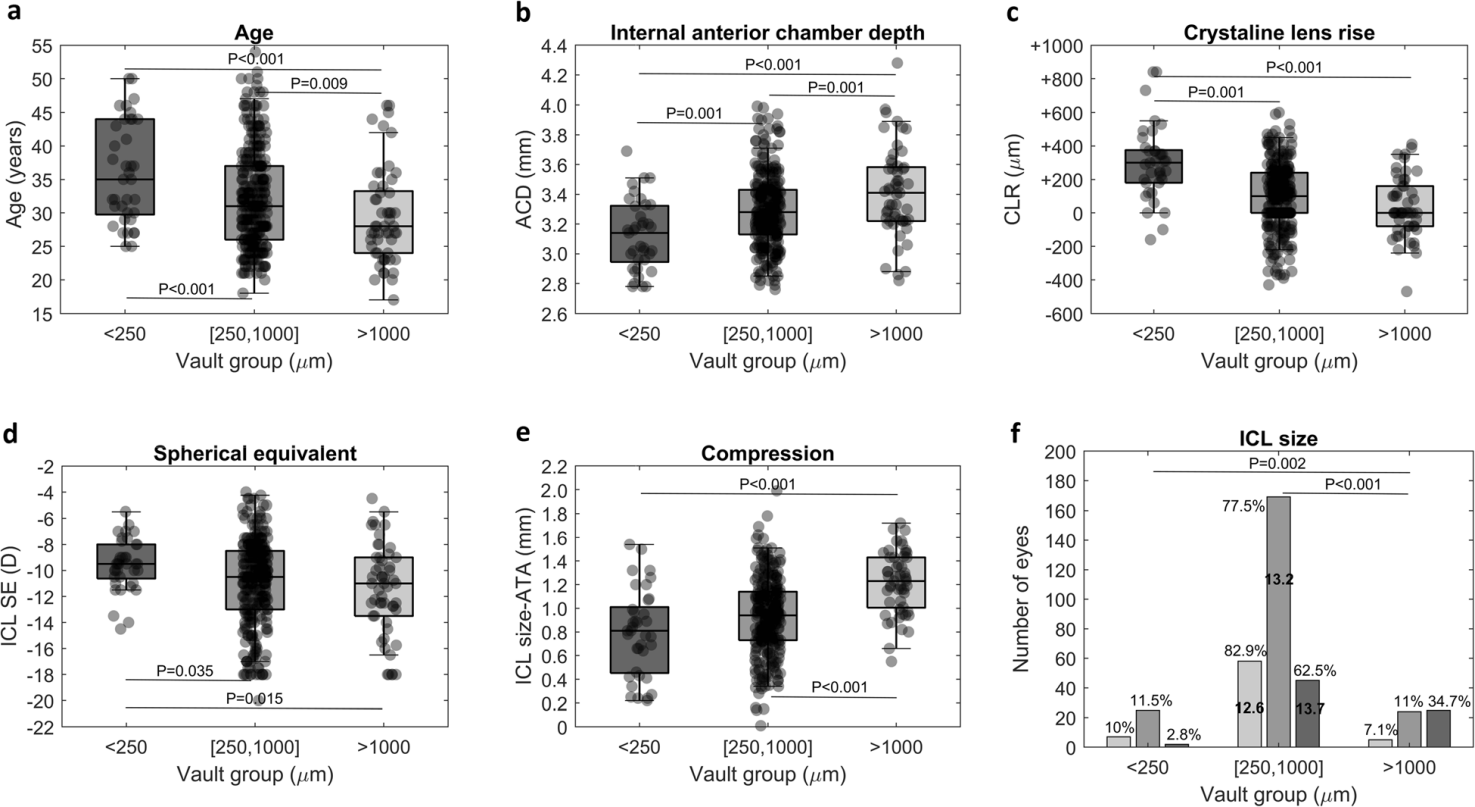
For the three vault groups: **a** Patient age in years; **b** Internal anterior chamber depth (ACD) in millimeters; **c** Crystalline lens rise (CLR) in micrometers; **d** ICL spherical equivalent (ICLSE) in diopters; **e** ICL size minus ATA (Compression) in millimeters; **f** Number of eyes in each vault group per ICL size (light, middle and dark shadows represent ICL size 12.6, 13.2 and 13.7 mm, respectively). Box limits = 25 and 75 percentiles, horizontal line within the box = median, and whiskers = 2.5 and 97.5 percentiles. In figure f the percentages indicate the proportion of eyes implanted with ICL 12.6, 13.2 and 13.7 mm in the three vault groups

For the variables showing statistically significant differences, pairwise comparisons indicated that older patients tended to have lower vaults (Fig. 1a) and eyes with deeper ACDs presented higher vaults (Fig. 1b). With regards to the CLR, eyes in the LVG had more protruded crystalline lenses compared to the eyes in the OVG and HVG (Fig. 1c). For the ICLSE, eyes in the LVG had on average less myopic ICLs compared to the remaining two groups (Fig. 1d). The ICL size-ATA, the indicator of the ICL compression was higher in the HVG compared to the LVG and OVG (Fig. 1e).

Concerning ICL size, there was a tendency for the 13.7 mm lens to present higher vaults compared to the 12.6 and 13.2 mm ICLs (Fig. 1d). Nearly 35% (*n* = 25) of the 13.7 mm ICLs implanted produced a vault higher than 1000 μm compared to the 7.1% (*n* = 5) and 11% (*n* = 24) produced by the 12.6 and 13.2 mm ICLs, respectively. Only 2.8% (*n* = 2) of the 13.7 mm ICLs produced vaults below 250 μm compared to 10% (*n* = 7) and 11.5% (*n* = 25) of the 12.6 and 13.2mm ICLs, respectively. The effect of ICL size on the vault magnitude can be associated to its size or to the amount of compression (difference between ICL size and ATA) induced by the ICL. An analysis of covariance (ANCOVA) between vault interval and ICL size using the compression as confounding factor confirmed the effect of ICL size on the vault F = 13.0, *P* < 0.001. ANCOVA post-hoc analysis revealed that significant differences occurred between ICLs of 13.7 and 12.6 mm (*P* < 0.001) and between 13.7 and 13.2 mm (*P* < 0.0001).

**Fig. 2:**
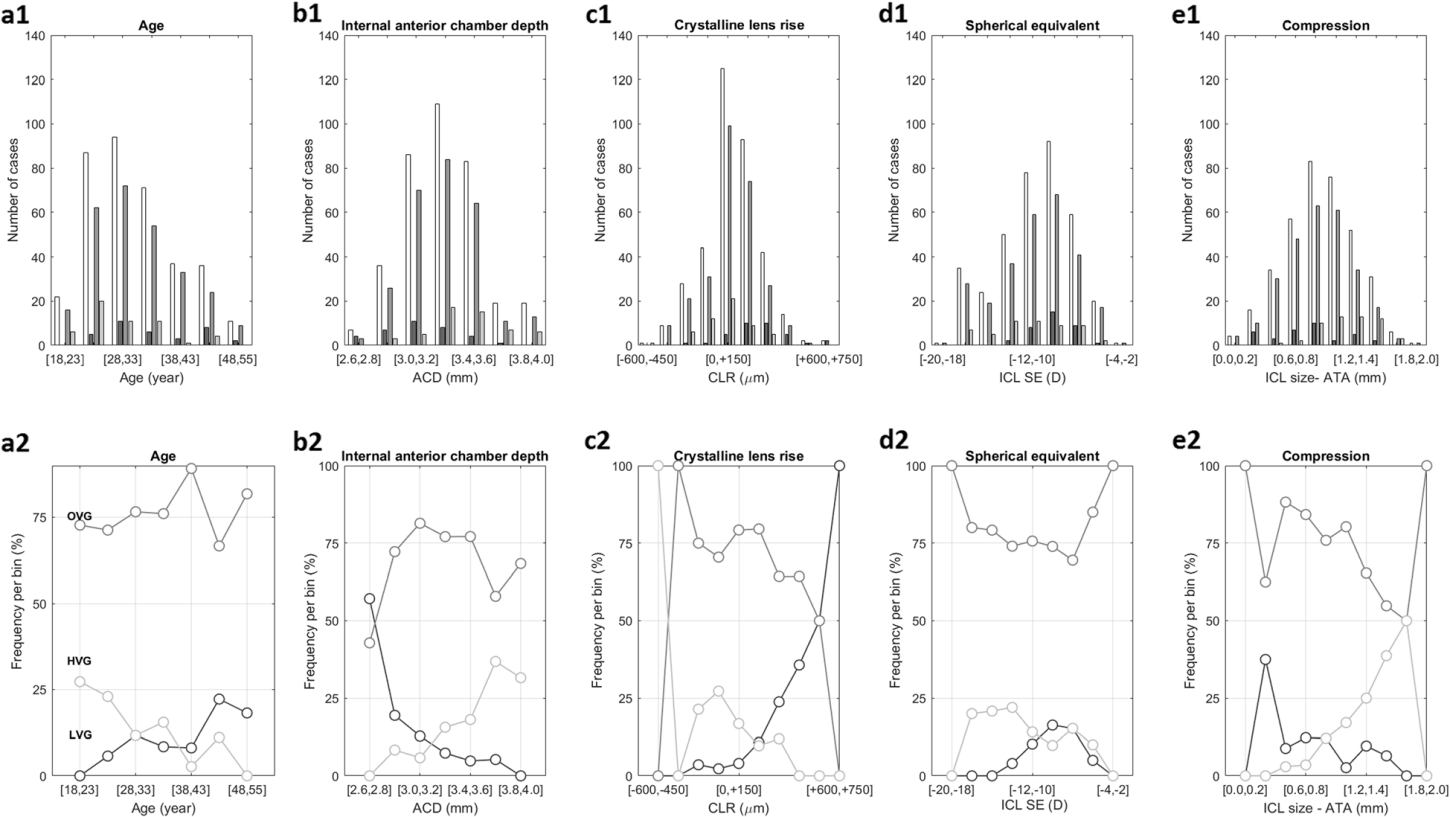
Upper graphs (**a1**-**e1**) show the distribution of the number of cases for **a1** Age; **b1** Internal anterior chamber depth (ACD); **c1** Crystalline lens rise (CLR); **d1** ICL spherical equivalent (ICLSE); **e1** ICL size minus ATA (Compression). White bars represent the total number of cases per bin; and dark, medium and light grey bars indicate the number of cases in the low, normal and high vault in each bin, respectively. Lower graphs (**a2**-**e2**) represent the frequency of cases in each bin, the dark, medium and light grey lines represent the low, normal and high vaults, respectively. For the sake of interpretation, considering the 18 to 23 year-old bin, there are 22 cases (0 low vault; 16 normal vault and 6 high vault). The frequency plot shows the dark grey line at 0, the medium grey at 72.7% and the light grey at 27.3%

Figure 2 (a1-e1) depicts the distribution of cases for those variables presenting statistically significant associations with the type of vault and on the lower row (a2-e2) the frequency of occurring a specific type of vault. The general picture shows that the majority of eyes had an optimal vault throughout the variables range of measurements, however the variations in the number of eyes with low and high vaults highlights the influence of each variable in the type of vault.

For instance, the frequency of low vaults increased from 4% for a CLR between 0 and 150 μm to 50% when the CLR was between 600 to 750 μm (Fig. 2-c2). For the difference between ICL size and ATA, the frequency of high vaults increased from 3.5% for a compression between 0.6 and 0.8 mm to 50% when the compression was between 1.6 and 1.8 mm (Fig. 2-e2). Deeper ACDs decreased the frequency of low vault from about 50% in narrow chambers (2.6 to 2.8 mm) to nearly 0% in larger ACDs whereas the frequency of high vaults increased from 0% to nearly 40% in larger ACDs (Fig. 2-b2). Regarding age and spherical equivalent, the changes in the frequency of optimal vault cases were less pronounced. The frequency of low vaults increased from 0% in the youngest patients to about 20% in the oldest patients and the converse is true for the HVG (Fig. 2-a2). In the higher spectrum of myopia, there was a higher number of cases with high vault, and in low myopias the number of cases with low vault tended to increase (Fig. 2-d2).

### Multinomial logistic regression

Stepwise multinomial logistic regression analysis used the preoperative anatomical biometry, ICL parameters and patient demographics to determine the influence of relevant variables on sub-optimal vaults. The model used the optimal vault range as reference and identified the CLR, ICLSE, Age, ICL size and ICL size-ATA (compression) as relevant factors for the presence of a low or high vault, final model χ^2^ (df = 16) = 129.7, *P* < 0.0001 with a pseudo-R^2^ (Nagelkerke) = 0.40 (Table 3).

**Table 3:** Multinomial logistic regression model summary

Variable	Low vault (*n* = 35)				High vault (*n* = 54)			
	Β_L_	OR	SE	OR 95% CI	β_H_	OR	SE	OR 95% CI
Constant	−1.401		1.221		−5.733			
Age (years)	0.028	1.028	0.026	0.98–1.08	−0.076	0.927	0.03	0.879–0.978 **
ICL size minus ATA (mm)	−0.506	0.603	0.628	0.176–2.065	3.721	41.287	0.70	10.574–161.218***
CLR (μm)	0.005	1.005	0.001	1.003–1.007***	0.000	1.000	0.89	0.998–1.002
ICLSE (DS)	0.202	1.223	0.070	1.067–1.402**	−0.164	0.848	0.06	0.760–0.948 **
ICL Size								
12.6 mm	0.142	1.153	0.500	0.433–3.069	−0.096	0.909	0.56	0.305–2.703
13.2 mm	0.000	1			0.000	1		
13.7 mm	−0.790	0.454	0.695	0.116–1.774	1.958	7.084	0.41	3.157–15.893**

Reference group: optimal vault (*n* = 218), β_L_(i) and β_H_(i): regression coefficients for low and high vault

*OR* odds ratio ; *SE* standard error; *CI* confidence interval; *ICL* implantable collamer lens; *ATA* horizontal anterior-chamber angle-to-angle distance; *CLR* crystalline lens rise; *ICLSE* implantable collamer lens spherical equivalent; *DS* diopter sphere

**P < 0.01; ***P < 0.001

With respect to the risk of developing low vaults, this increased in eyes with a higher CLR (OR: 1.005) and lower myopic (i.e., less negative dioptric power) ICLSE (OR: 1.223). As far as the risk of high vault is concerned, eyes with higher compression (OR: 41.287) and higher myopic spherical equivalent (SE) (OR: 0.848) had a higher chance of presenting high vaults. Older age (OR: 0.927) and less myopic SE (OR: 0.848) were protective factors for presenting a high vault. In relation to the ICL size, patients implanted with 13.7 mm ICLs (OR = 7.084) were seven times more likely to present a high vault compared to patients implanted with 13.2 mm ICLs.

**Fig. 3:**
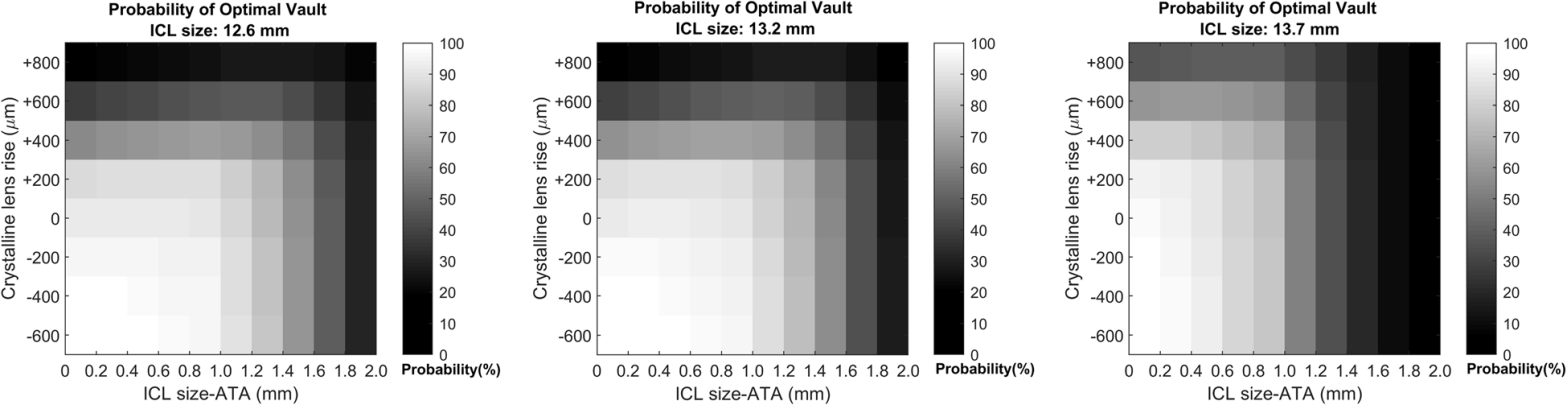
Probability of having an optimal vault for three ICL sizes (12.6, 13.2 and 13.7 mm). The probability, derived from the multinomial regression, for a 32 year-old patient with a − 10.00 DS equivalent. The horizontal axis represents the variation in compression (i.e., ICL size minus ATA) and the vertical axis the crystalline lens rise. Considering the grey scale, white stands for the highest probability and black for the lowest

The probability of an optimal vault (range: 250–1000 μm) can be determined for the 12.6, 13.2 and 13.7 mm ICLs by varying the CLR and ICL size-ATA and constraining the parameter patient age and ICLSE (details in Appendix). Figure 3 shows the full range of probabilities expected for a 32 year-old patient with − 10.00 DS ICLSE.

## Discussion

When selecting the size of an ICL, the clinician has to decide most times between two of the four available sizes and should select the one that offers the highest probability of obtaining an optimal vault. In this study, we assessed the influence of biometric and ICL-related parameters associated with sub-optimal vaults. The results demonstrated that sub-optimal vaults were associated with a series of parameters namely, ICL compression, crystalline lens anterior protrusion, ICL size, ICL spherical power and patient’s age. However, the influence of each of these factors depended on the type of sub-optimal vault. The risk of low vaulting increased in eyes with more protruded crystalline lens and low myopic ICL power, whereas the risk of high vaulting was aggravated by an excessive compression, in particular for the 13.7 mm ICL, a high myopic ICL power and younger age.

The influence of crystalline lens protrusion in the vault has been reported in various studies [21, 24, 25, 28, 29]. Kojima et al. using ultrasound biomicroscopy (UBM) reported that the distance separating the sulcus-to-sulcus (STS) line from the anterior apex of the crystalline lens (a similar measurement to the CLR used in this study) ranged in their study’s sample between − 40 to + 690 μm, representing a dimensional amplitude relevant to the vault determination [28]. Zheng et al. and Qi et al. respectively reported that the anterior surface curvature and thickness of the crystalline lens were morphologic features associated with the vault [21, 29]. In a study analyzing the vault’s dynamic behavior, Gonzalez-Lopez et al. reported that eyes with high vaults (> 750 μm in mydriasis) had lower CLRs (+ 73 μm) compared to those (+ 352 μm) with low vault (< 100 μm in miosis) [25]. Recently, two vault prediction formulas based on AS-OCT imaging predicted a decrease in vault between 37 to 40 μm per 100 μm of CLR increase [7, 24]. Considering the CLR range found in this study (95% CI: − 240 to + 450 μm), the CLR per se may account for differences in vault as high as 280 μm. Our results show an important association between the CLR and the presence of low vaulting. The odds of presenting a vault ≤ 250 μm increased by 0.5% per μm of CLR and the number of eyes with low vault increased gradually for those with CLRs above 150 μm, indicating that high protrusion of the anterior crystalline lens, here represented by the CLR, is the main factor contributing towards low vaulting.

The difference between the transverse size of the eye, measured either by the STS, ATA or WTW has been the most common parameter associated with the vault [7, 19–21, 23, 24, 28]. Lee et al. reported a positive correlation between the vault and compression of the ICL, suggesting that the compression created by an oversized ICL was the main regulator of the vault [20]. Later, Reinstein et al. proposed a geometrical model based on UBM measurements where the forward bulging of the ICL, therefore the vault, could be predicted by the difference between the ICL size and the STS [5]. Vault prediction formulas based on AS-OCT imaging, predict for each 0.1 mm increase in compression, an increase in vault between 75 to 30 μm [7, 23, 24]. The present results show that the main risk factor for a high vault is the difference between the ICL size and the ATA (OR: 41.29) i.e., an oversized ICL. For differences above 1.0 mm, there was a gradual increase in the number of eyes with high vault. Nearly 75% of the eyes with high vault had a compression higher than 1.05 mm contrasting with the 25% in the optimal vault group. Conversely, the results did not show strong evidence that low vaulting was associated with low compression. This observation is in tandem with previous evidence where ICLs implanted with low compression yielded optimal vaults [20]. Nam et al. suggested the presence of a buffering zone, associated with the intrinsic sagittal depth of the ICL, to justify optimal vaulting in eyes with undersized ICLs [26].

ICL size was another factor associated with high vaulting. In particular, eyes implanted with 13.7 mm ICL were seven times (OR: 7.08) more likely to present high vaults compared to eyes implanted with 13.2 mm ICL. This was related to a higher effect of the compression in the 13.7 mm ICL compared to that in the 13.2 and 12.6 mm ICLs, rather than differences in the compression among the three ICL sizes. A study by our group found a mean variation in vault of 53 μm per 0.1 mm of compression with the 13.7 mm ICL compared to 32 μm for the 12.6 and 13.2 mm sizes [24]. Applying the model presented here, to maintain the probability of occurring a high vault below 10%, the minimum ATA distance to consider for the 12.6, 13.2 and 13.7 mm should be 11.4, 12.1 and 13.0 mm, respectively.

The dioptric power of the ICL was another factor associated with sub-optimal vaults, while low myopic ICLs represented a risk factor for low vaulting it was a protective factor for high vaulting. The intrinsic sagittal depth of an ICL depends on its dioptric power; for the ICL-V4 model the sagittal depth raises from 1.04 to 1.94 mm for a dioptric interval between − 3.00 to − 23.00 DS [20]. Regression formulas using ICL power as vault predictor estimate 20 to 25 μm increase in vault per diopter of myopic power, representing a variation in the vault of approximately 350 μm attributable to a ICL power range between − 2.00 to − 18.0 DS.

Older patient age at the time of surgery was identified as a protective factor for high vaulting with the risk reducing 7% per year of age. Previous reports found a reduction in the vault of approximately 5 μm per year of age [24, 30]. We believe that the effect of age in the vault was mainly associated with the increase of the crystalline lens thickness with age [31] and affected the vault in similar manner to what was previously described for the CLR. Interestingly, no statistically significant association was found between age and the incidence of low vaults despite the fact that increasing age points towards a higher risk of low vaulting. We believe that the poor statistical relationship between CLR and age (*R*^*2*^ = 0.08, data not shown) might have contributed to this association. A second contributor associated with age is the well-known reduction in pupil size with aging [32], which may increase the anterior-posterior force applied by the pupil on the ICL, thereby reducing the vault [13]. However, the pupil size was not controlled in this study and future studies are required to test this hypothesis.

In this case series, all ICL sizes were selected according to the manufacturer’s calculator and 25% of the cases had vaults outside the 250–1000 μm range, concurring with previous reports [26]. Neither variables (ICL size-WTW and ACD) used by the manufacturer to indicate the ICL size were associated with sub-optimal vaults. The reasons may be, the WTW being a structure poorly correlated with the STS [33] not representing accurately the transverse size of the eye and the anterior chamber depth not being a direct regulator of the vault [25]. Alternative parameters, based on AS-OCT imaging, such as the ATA and the CLR are more useful biometric parameters at the time of selecting the most adequate ICL size.

One of the limitations of this study is the number of cases in the sub-optimal vault groups and in the boundaries of the predictor variables. Future studies including more data may improve the accuracy of these models. Despite this, the model was able to not only trace associations directly related to the vault mechanism but also trace indirect associations (e.g., ICLSE). This suggests that the sample size provided enough power for the predictions. Another limitation is the fact that ICL vaulting has an important dependency on the haptics position [6], and thus an analysis of the ICL resting position using UBM could be used to improve the model. Changes in the anterior segment anatomy induced by ambient-light conditions have been demonstrated to influence the vault and their introduction in prediction models may improve the vault prediction ability [25]. In the present study, we assume that although not controlled accurately, the variations in the lighting conditions varied minimally since the same room and ambient light was used, and therefore the results should be interpreted in accordance with the measuring conditions used. Furthermore, we did not distinguish between eyes implanted with spherical and toric ICLs. However, we believe that the nature of the ICL is similar between both designs and important differences between vault predictors are not expected between both ICLs.

## Conclusions

In conclusion, this study demonstrates that using anterior segment biometric measurements such as those obtained with the AS-OCT is possible to control parameters associated with sub-optimal vaults. The novelty of this work lies in the fact that parameters associated with the vault have a different weight depending on whether the sub-optimal vault is low or high. Furthermore, we provide a hierarchical approach based on primary (CLR and ICL size-ATA/ ICL size) and secondary risk factors (ICLSE and age) that may assist the clinician in selecting the most suitable ICL size.

### On-line resources

The authors provide here (https://ruipinge.github.io/icl-calc/) an online calculator to estimate the probability of a safety vault using the equations described in the Additional file 1.

## Supplementary Information

**Additional file 1**

## Data Availability

The data used in this study can be requested by contacting the corresponding author.
