# Supplementary material for: Biometric and ICL-related risk factors associated to sub-optimal vaults in eyes implanted with implantable collamer lenses
Source: Eye Vis (Lond). 2021 Jul 5;8:26. doi: 10.1186/s40662-021-00250-6 (PMC8256545; doi:10.1186/s40662-021-00250-6)
Supplement: Supplementary file 1 — Additional file 1 [file 40662_2021_250_MOESM1_ESM.docx]

**Appendix**

Using a binary logistic function, the probability (P) a specific event occurring based on a set of independent variables (X) can be described,

| $ln\left\lceil\frac{P}{1-P} \right\rceil=\beta_{0}+\beta_{1}X_{1}+\beta_{2}X_{2}+\ldots$ | Eq. 1 |
| --- | --- |

Where βn (n>1) represents the slopes of the independent variables and β_0_ the interception of the best fit curve.

Solving equation 1, the probabilities of an event and of a non-event occurring are given by,

| $P(event)=\frac{e^{\beta_{0}+\beta_{1}X_{1}+\beta_{2}X_{2}+\ldots}}{1+e^{\beta_{0}+\beta_{1}X_{1}+\beta_{2}X_{2}+\ldots}}$ | Eq. 2 | $P(\sim event)=\frac{1}{1+e^{\beta_{0}+\beta_{1}X_{1}+\beta_{2}X_{2}+\ldots}}$ | Eq. 3 |
| --- | --- | --- | --- |

In cases where the nominal dependent variables have two or more levels, multinomial regression analysis equation 1 can be rewritten as,

| $ln\left\lceil\frac{P_{Event A}}{1-P_{Event A}} \right\rceil=\beta_{0A}+\beta_{1A}X_{1}+\beta_{2A}X_{2}+\ldots$ | Eq. 4 |
| --- | --- |

| $ln\left\lceil\frac{P_{Event B}}{1-P_{Event B}} \right\rceil=\beta_{0B}+\beta_{1B}X_{1}+\beta_{2B}X_{2}+\ldots$ | Eq. 5 |
| --- | --- |

The probability equations for the three levels of dependent variables are

| $P(event A)=\frac{e^{\beta_{0A}+\beta_{1A}X_{1}+\beta_{2A}X_{2}+\ldots}}{1+e^{\beta_{0A}+\beta_{1A}X_{1}+\beta_{2A}X_{2}+\ldots}+e^{\beta_{0B}+\beta_{1B}X_{1}+\beta_{2B}X_{2}+\ldots}}$ | Eq. 6 |
| --- | --- |

| $P(event B)=\frac{e^{\beta_{0B}+\beta_{1B}X_{1}+\beta_{2B}X_{2}+\ldots}}{1+e^{\beta_{0A}+\beta_{1A}X_{1}+\beta_{2A}X_{2}+\ldots}+e^{\beta_{0B}+\beta_{1B}X_{1}+\beta_{2B}X_{2}+\ldots}}$ | Eq. 7 |
| --- | --- |

| $P(\sim(event A or event B))=\frac{1}{1+e^{\beta_{0A}+\beta_{1A}X_{1}+\beta_{2A}X_{2}+\ldots}+e^{\beta_{0B}+\beta_{1B}X_{1}+\beta_{2B}X_{2}+\ldots}}$ | Eq. 8 |
| --- | --- |

The probabilities of a low, optimal and high vault can be computed using the coefficients Age, ICL size - ATA (Compression), CLR, ICLSE and ICL size from Table 3 and are described in equations 9-11.

| $P("Low Vault")=\frac{exp(B_{Lens, Low}+0.028 Age-0.506 Compression+0.005 CLR+0.202 ICLSE)}{1+exp(B_{Lens, Low}+0.028 Age-0.506 Compression+0.005 CLR+0.202 ICLSE)+exp(B_{Lens, High}-0.076 Age+3.721 Compression+0.0 CLR-0.164 ICLSE)}$ | Eq. 9 |
| --- | --- |

| $P("Optimal Vault")=\frac{1}{1+exp(B_{Lens, Low}+0.028 Age-0.506 Compression+0.005 CLR+0.202 ICLSE)+exp(B_{Lens, High}-0.076 Age+3.721 Compression+0.0 CLR-0.164 ICLSE)}$ | Eq. 10 |
| --- | --- |

| $P("High Vault")=\frac{exp(B_{Lens, High}-0.076 Age+3.721 Compression+0.0 CLR-0.164 ICLSE)}{1+exp(B_{Lens, Low}+0.028 Age-0.506 Compression+0.005 CLR+0.202 ICLSE)+exp(B_{Lens, High}-0.076 Age+3.721 Compression+0.0 CLR-0.164 ICLSE)}$ | Eq. 11 |
| --- | --- |

Where B_Lens_ represents the constants for the 12.6, 13.2 and 13.7 lenses, such as B_12.6,_ _Low_=−1.259, B_13.2,_ _Low_=−1.401_,_ B_13.7,_ _Low_=−2.191, and B_12.6,_ _High_=−5.829, B_13.2,_ _High_ =−5.733, B_13.7,_ _High_ =−3.775.
